# Amrubicin-Induced Radiation Recall Pneumonitis After Low-Dose Palliative Thoracic Radiotherapy: A Case Report

**DOI:** 10.7759/cureus.117848

**Published:** 2026-10-13

**Authors:** Yuya Fujii, Kaisei Iwamoto, Ryosuke Kojima, Shinichi Nakanishi, Riki Matsukawa

**Affiliations:** 1 Department of Respiratory Internal Medicine, Fukui Prefectural Hospital, Fukui, JPN

**Keywords:** amrubicin, drug-induced lung injury, palliative radiotherapy, radiation recall pneumonitis, small-cell lung cancer

## Abstract

A 70-year-old man with extensive-stage small-cell lung cancer received palliative thoracic radiotherapy to a total dose of 18 Gy in six fractions for central airway stenosis. Five months later, second-line amrubicin was initiated. After two courses of treatment, he was admitted with fever and cough and was initially treated for suspected bacterial pneumonia. Despite cefepime treatment, computed tomography revealed worsening pulmonary opacities corresponding to the previously irradiated field. Bronchoscopy showed no visible endobronchial tumor, cytology revealed no malignant cells, and microbiological studies were negative. Amrubicin-induced radiation recall pneumonitis (RRP) was clinically diagnosed. Amrubicin was discontinued, and oral prednisolone was initiated at 30 mg/day, resulting in rapid clinical and radiographic improvement. Prednisolone was subsequently tapered to 10 mg/day without radiographic worsening at that dose. This case highlights that RRP should be considered after amrubicin therapy, even when the previous thoracic radiation dose was relatively low.

## Introduction

Radiation recall pneumonitis (RRP) is an uncommon inflammatory reaction that occurs in previously irradiated lung tissue after administration of a triggering agent [1]. Various systemic treatments, including cytotoxic chemotherapy, molecularly targeted therapy, and immune checkpoint inhibitors, have been implicated in RRP [1,2]. In a representative series of chemotherapy-induced RRP after thoracic radiotherapy for lung cancer, the causative agents included taxanes, etoposide, vinorelbine, and epirubicin, but not amrubicin [3]. Detailed clinical information on amrubicin-induced RRP remains limited, particularly in the setting of low-dose palliative thoracic radiotherapy for small-cell lung cancer.

In this report, we present a case of clinically diagnosed RRP after second-line amrubicin therapy in a patient with extensive-stage small-cell lung cancer who previously received palliative thoracic radiotherapy to a total dose of 18 Gy.

## Case presentation

A 70-year-old man with extensive-stage small-cell lung cancer underwent palliative thoracic radiotherapy for central airway stenosis in October 2025. Although the initial treatment plan prescribed 30 Gy in 10 fractions, radiotherapy was terminated after six fractions following clinical improvement, resulting in a total delivered dose of 18 Gy. The clinical target volume was 665.8 mL, and the estimated cumulative mean lung dose was 5.82 Gy, calculated from the original treatment plan according to the number of fractions delivered.

Disease progression was subsequently observed, and second-line chemotherapy with amrubicin (35 mg/m²/day intravenously on days 1-3 of each 21-day cycle) was initiated in March 2026.

After two courses of amrubicin, the patient was admitted in May 2026 with fever and cough and was initially suspected to have pneumonia involving the lingular segment. Cefepime was initiated for presumed bacterial pneumonia; however, his clinical condition did not improve sufficiently. Laboratory findings on admission are summarized in Table 1. C-reactive protein and the interstitial lung disease markers Krebs von den Lungen-6 and surfactant protein-D were elevated, whereas the autoimmune serological tests were negative.

**Table 1:** Laboratory Findings on Admission ANA, antinuclear antibody; ANCA, antineutrophil cytoplasmic antibody; CCP, cyclic citrullinated peptide; CMV, cytomegalovirus; ILD, interstitial lung disease; MPO, myeloperoxidase; PCR, polymerase chain reaction; PR3, proteinase 3; SP-D, surfactant protein-D; SS-A/Ro, Sjögren syndrome-related antigen A/Ro

Category	Parameter	Result	Unit	Reference range
Hematology	Red blood cell count (RBC)	3.45	×10⁶/μL	4.35-5.55
	Hemoglobin (Hb)	10.7	g/dL	13.7-16.8
	Hematocrit (Ht)	33.7	%	40.7-50.1
	Platelet count (PLT)	190	×10³/μL	158-348
	White blood cell count (WBC)	4.1	×10³/μL	3.3-8.6
	Neutrophils	58.2	%	45.2-68.8
	Eosinophils	3.2	%	0.0-10.0
	Basophils	1.2	%	0.0-5.0
	Monocytes	16.5	%	2.7-7.9
	Lymphocytes	20.9	%	26.8-43.8
Biochemistry	Total protein (TP)	6.8	g/dL	6.6-8.1
	Albumin (Alb)	3.4	g/dL	4.1-5.1
	Lactate dehydrogenase (LDH)	209	U/L	124-222
	Blood urea nitrogen (BUN)	4.1	mg/dL	8-20
	Creatinine (Cre)	0.88	mg/dL	0.65-1.07
	Sodium (Na)	137	mmol/L	138-145
	Potassium (K)	4.1	mmol/L	3.6-4.8
	Creatine kinase (CK)	42	U/L	59-248
	C-reactive protein (CRP)	2.48	mg/dL	≤0.14
Immunology / ILD markers	Rheumatoid factor (RF)	5	IU/mL	≤15
	Anti-SS-A/Ro antibody	<0.1	IU/mL	<10.0
	Anti-CCP antibody	<0.06	IU/mL	<4.5
	PR3-ANCA	<0.6	IU/mL	<2.0
	MPO-ANCA	<0.2	IU/mL	<3.5
	Antinuclear antibody (ANA)	<1:40	titer	<1:40
	Krebs von den Lungen-6 (KL-6)	507	U/mL	<500
	Surfactant protein-D (SP-D)	135	ng/mL	<110
Infection-related tests	β-D-glucan	5.7	pg/mL	<11
	Cytomegalovirus PCR	Negative	-	Negative

Chest computed tomography (CT) before amrubicin therapy showed no apparent active pneumonitis in the irradiated field on axial and coronal images (Figures 1A, 1C). However, CT after admission showed worsening pulmonary opacities along the previously irradiated field on axial and coronal images (Figures 1B, 1D). Comparison of the CT images (Figures 2A, 2C) with the corresponding radiation dose distribution maps (Figures 2B, 2D) demonstrated that the pulmonary opacities corresponded to the previous radiation field.

**Figure 1:**
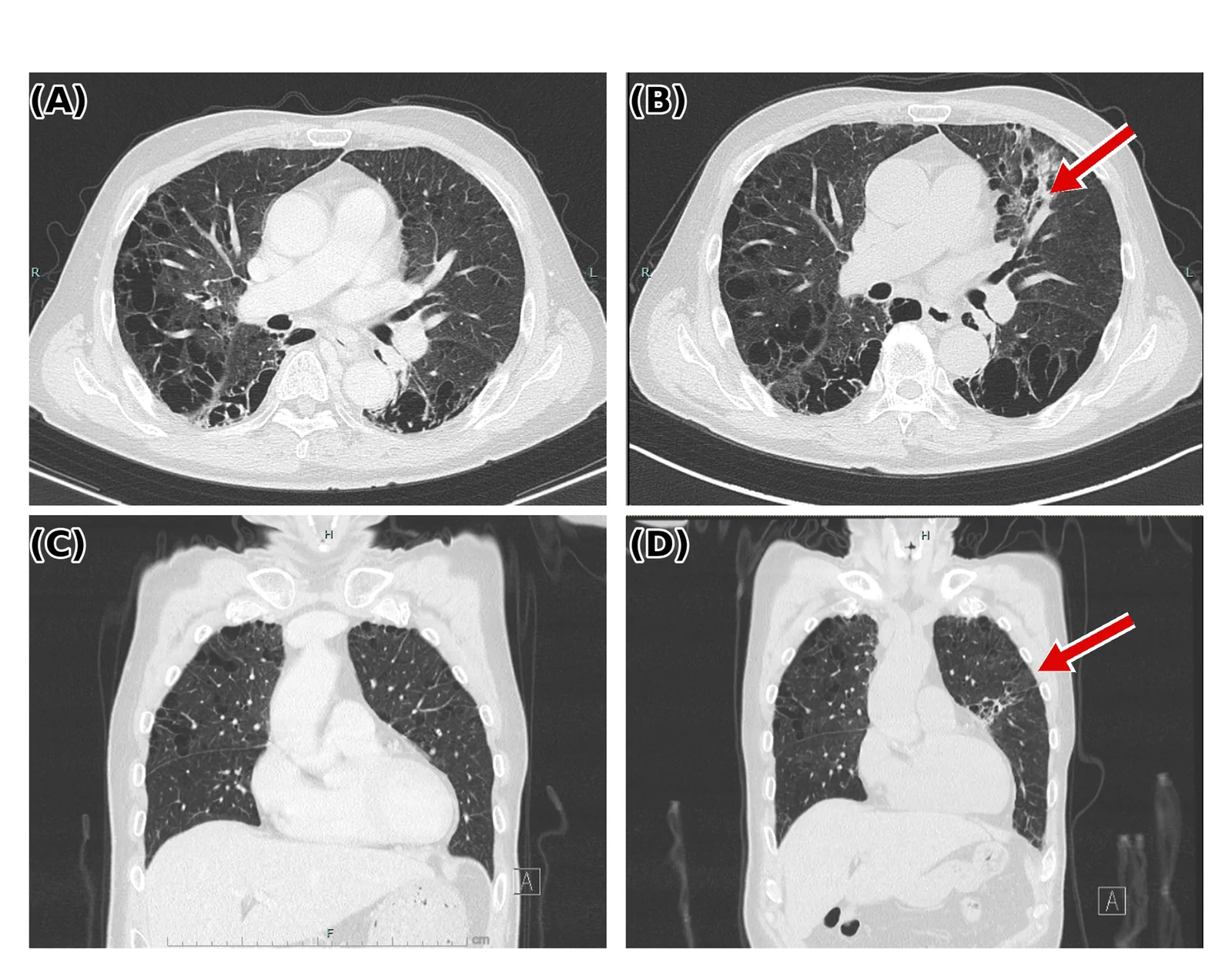
Chest Computed Tomography Images Before and at the Onset of Radiation Recall Pneumonitis (A) Axial chest computed tomography before amrubicin therapy, showing no apparent active pneumonitis in the irradiated field. (B) Axial chest computed tomography at the onset of pneumonitis, showing new pulmonary opacities corresponding to the previously irradiated field (arrow). (C) Coronal chest computed tomography before amrubicin therapy, showing no apparent active pneumonitis in the irradiated field. (D) Coronal chest computed tomography at the onset of pneumonitis, showing new pulmonary opacities corresponding to the previously irradiated field (arrow).

**Figure 2:**
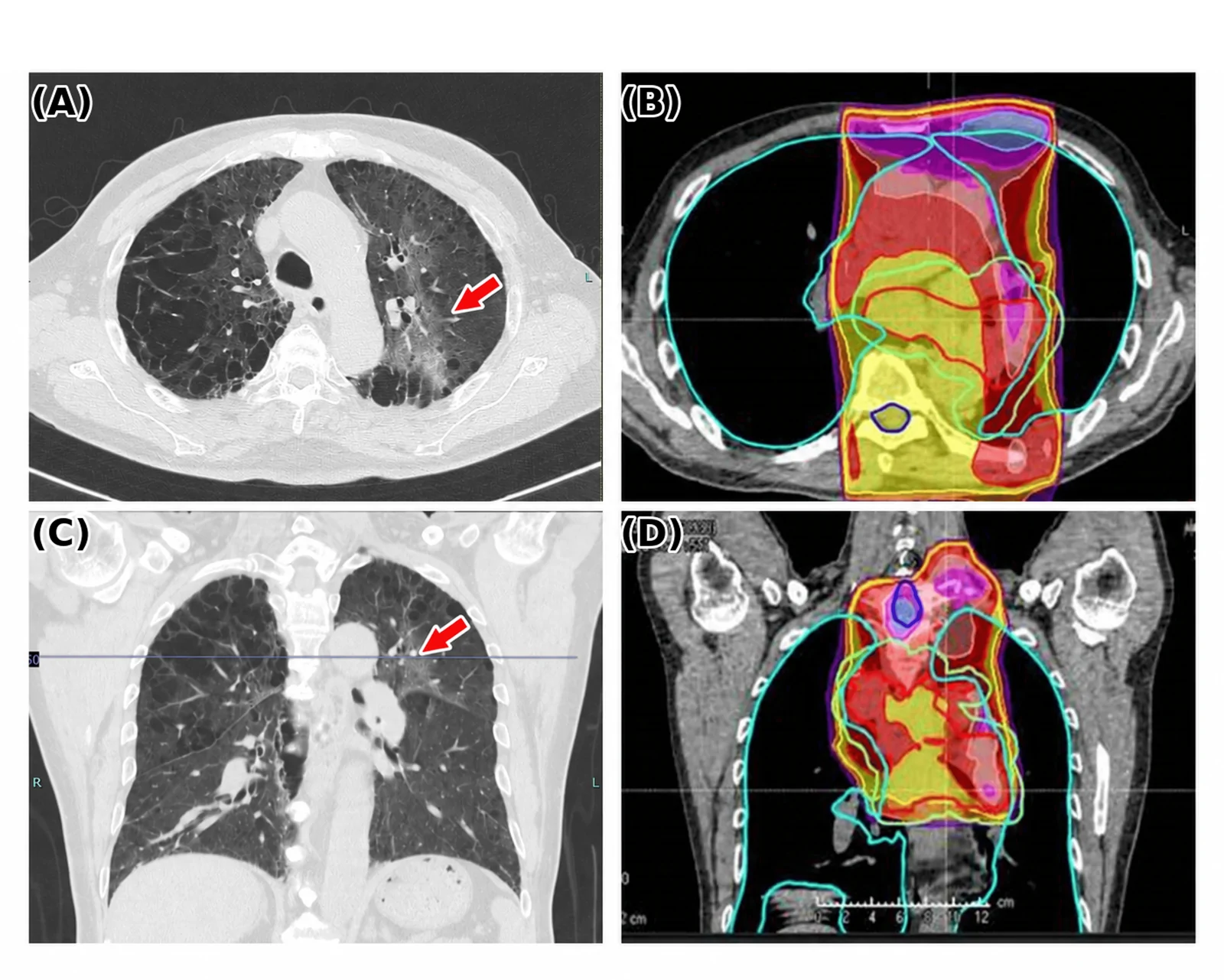
Relationship Between Worsening Pulmonary Opacities and the Previous Radiation Field (A) Axial chest computed tomography after worsening of pulmonary opacities; the arrow indicates the pulmonary opacity. (B) Corresponding axial radiation dose distribution map showing that the pulmonary opacity lies within the previously irradiated field. (C) Coronal chest computed tomography after worsening of pulmonary opacities; the arrow indicates the pulmonary opacity. (D) Corresponding coronal radiation dose distribution map showing that the pulmonary opacity lies within the previously irradiated field.

Infectious pneumonia, tumor progression, amrubicin-induced lung injury, and conventional radiation pneumonitis were considered in the differential diagnosis. Bronchoscopy revealed no visible endobronchial tumors. Bronchoalveolar lavage (BAL) fluid showed a total cell count of 522/μL, with 75% lymphocytes. Cytology revealed no malignant cells. Bacterial and mycobacterial cultures of BAL fluid and airway specimens were negative. Serum β-D-glucan and cytomegalovirus polymerase chain reaction testing were also negative.

Based on newly worsened field-conforming pulmonary opacities after amrubicin therapy, antibiotic refractoriness, negative cytology, and the absence of microbiological evidence of infection, amrubicin-induced RRP was clinically diagnosed. Amrubicin was discontinued, and oral prednisolone was initiated at 30 mg/day. The patient's fever, cough, and radiographic findings improved rapidly. The initial prednisolone dose of 30 mg/day was maintained for two weeks, followed by a gradual taper of 5 mg every two weeks. The prednisolone dose was subsequently reduced to 10 mg/day without radiographic worsening of the pulmonary opacities at that time.

## Discussion

This case illustrates a clinically important diagnostic pitfall in the management of small-cell lung cancer. Amrubicin is used for relapsed small-cell lung cancer but has been associated with interstitial lung disease [4]. In a representative chemotherapy-induced RRP series by Ding et al., the causative agents included taxanes, etoposide, vinorelbine, and epirubicin, but not amrubicin [3]. The present case provides clinically useful information for the diagnosis and management of suspected amrubicin-induced RRP, including serial CT findings, comparison with radiation dose distribution, BAL findings, negative microbiological and cytological evaluations, and corticosteroid responsiveness.

The precise pathophysiological mechanism of RRP remains unclear. Several mechanisms have been proposed, including radiation-induced depletion of tissue stem or progenitor cells, alterations in local vascular and immune responses, and increased sensitivity of previously irradiated tissue to subsequent drug exposure [1,2]. Persistent changes in the local inflammatory microenvironment may also lower the threshold for drug-triggered inflammatory responses through cytokine-mediated mechanisms [1,2]. In the present case, prior thoracic irradiation may have increased the susceptibility of the affected lung tissue to amrubicin-associated inflammation, even after a relatively low total delivered radiation dose of 18 Gy. However, the specific mechanism underlying amrubicin-induced RRP remains speculative.

RRP developed after palliative thoracic radiotherapy to a relatively low total dose of 18 Gy in six fractions. The estimated cumulative mean lung dose was 5.82 Gy. Reports on RRP after low-dose or palliative thoracic radiotherapy are limited. In the series by Ding et al., which included 12 patients with chemotherapy-induced RRP after thoracic radiotherapy for lung cancer, the radiation dose ranged from 52 to 66 Gy, with a median dose of 60.7 Gy [3]. Only a few English-language reports have described RRP after palliative thoracic radiotherapy, mostly associated with epidermal growth factor receptor tyrosine kinase inhibitors. Awad and Nott reported erlotinib-induced RRP after palliative thoracic radiotherapy for lung cancer [5]. Onal et al. also reported erlotinib-induced RRP after palliative hypofractionated thoracic radiotherapy, with a lung V20 of 21.2% and a mean lung dose of 12.7 Gy [6]. In contrast, the present patient developed RRP after an actual delivered radiation dose of 18 Gy and subsequent amrubicin therapy. This case suggests that RRP should not be excluded solely because the previous thoracic radiation dose was low when opacities conform to the irradiated field after administration of a potential triggering agent.

The diagnosis of RRP in the present case was supported by the field-conforming distribution of pulmonary opacities, temporal relationship with amrubicin administration, antibiotic refractoriness, negative cytology and microbiological findings, and rapid improvement with corticosteroid therapy. The timing of RRP may overlap with that of conventional radiation pneumonitis; therefore, timing alone is insufficient to distinguish these entities. Conventional radiation pneumonitis generally develops within weeks to months after thoracic radiotherapy and usually manifests within one to six months [7]. In a representative series of chemotherapy-induced RRP, the median interval from completion of radiotherapy to RRP was 95 days, and the median interval from chemotherapy initiation to RRP was 47 days, with a range of 22-169 days [3]. Therefore, the onset after two courses of amrubicin in the present case was compatible with the reported timing of chemotherapy-induced RRP. In such cases, diagnosis should consider whether pulmonary opacities newly appear or worsen after administration of a potential triggering agent, whether they conform to the previously irradiated field, whether pre-trigger CT shows stable or absent pneumonitis, and whether infection and tumor progression have been reasonably excluded [2,8]. In the present case, CT before amrubicin therapy showed no apparent active pneumonitis in the irradiated field, whereas the opacities worsened after two courses of amrubicin, corresponding to the previous radiation field. These findings support RRP rather than conventional radiation pneumonitis.

The potential association between prior thoracic irradiation and amrubicin-associated pulmonary toxicity has also been suggested by clinical trial data. In the JCOG0901 phase II study of amrubicin monotherapy for refractory small-cell lung cancer, pneumonitis occurred in five of 13 patients (38.5%) with a history of thoracic radiotherapy, compared with four of 69 patients (5.8%) without such a history [9]. Although these events were not specifically classified as RRP, and the subgroup sizes were small, this observation suggests a potential association between previous thoracic irradiation and subsequent amrubicin-associated pulmonary injury. The field-conforming distribution of pulmonary opacities in the present case provides additional clinical support for a possible radiation recall phenomenon.

Amrubicin-induced lung injury was also an important differential diagnosis. This represents an important limitation because RRP was clinically diagnosed without histopathological confirmation, and an overlap between RRP and drug-induced lung injury cannot be completely excluded. However, in the present case, the opacities were not diffuse but corresponded to the previously irradiated field, supporting RRP.

The main treatment for RRP consists of discontinuation of the suspected causative agent, corticosteroid therapy, and supportive care [1]. The patient improved rapidly after discontinuation of amrubicin and initiation of prednisolone. Prednisolone was subsequently tapered from 30 mg/day to 10 mg/day without radiographic worsening at that dose. Rechallenge should be considered cautiously because amrubicin itself can cause interstitial lung disease [4].

## Conclusions

This case describes clinically diagnosed amrubicin-induced RRP after low-dose palliative thoracic radiotherapy for extensive-stage small-cell lung cancer. The development of pulmonary opacities corresponding to the previously irradiated field after amrubicin administration, together with negative microbiological and cytological findings and rapid improvement after drug discontinuation and corticosteroid treatment, supported the diagnosis.

Although conventional radiation pneumonitis and amrubicin-induced lung injury can be difficult to distinguish from RRP, clinicians should consider radiation recall in patients who develop new or worsening field-conforming pulmonary opacities after amrubicin therapy. Importantly, a relatively low previous thoracic radiation dose does not exclude the possibility of RRP.
